# Dual phylogenetic origins of Nigerian lions (*Panthera leo*)

**DOI:** 10.1002/ece3.1116

**Published:** 2014-06-11

**Authors:** Talatu Tende, Staffan Bensch, Ulf Ottosson, Bengt Hansson

**Affiliations:** 1 A. P. Leventis Ornithological Research InstituteP.O. Box 13404, Jos, Nigeria; 2 Department of Biology, Lund UniversityEcology Building, Lund, SE-223 62, Sweden

**Keywords:** Dual origins, fecal DNA, Nigerian lions, Sanger sequencing

## Abstract

Lion fecal DNA extracts from four individuals each from Yankari Game Reserve and Kainji-Lake National Park (central northeast and west Nigeria, respectively) were Sanger-sequenced for the mitochondrial cytochrome *b* gene. The sequences were aligned against 61 lion reference sequences from other parts of Africa and India. The sequence data were analyzed further for the construction of phylogenetic trees using the maximum-likelihood approach to depict phylogenetic patterns of distribution among sequences. Our results show that Nigerian lions grouped together with lions from West and Central Africa. At the smaller geographical scale, lions from Kainji-Lake National Park in western Nigeria grouped with lions from Benin (located west of Nigeria), whereas lions from Yankari Game Reserve in central northeastern Nigeria grouped with the lion populations in Cameroon (located east of Nigeria). The finding that the two remaining lion populations in Nigeria have different phylogenetic origins is an important aspect to consider in future decisions regarding management and conservation of rapidly shrinking lion populations in West Africa.

## Introduction

There have been recent reports about a rapid reduction in population size and range distribution of lions (Estes et al. 2011; Packer et al. 2013). Until the end of the last glaciation period, lions were broadly distributed and roamed most parts of southern Europe, Asia, the Middle East, North America, northern part of South America, and sub-Saharan Africa (Coheleach 1982; Turner and Anton 1997; Bauer and Vander Merwe 2004; Werdelin and Lewis 2005). They were believed to have had the widest geographical distributions of any large terrestrial mammal in the late Pleistocene (Guthrie 1990; Kitchener 1991; Nowell and Jackson 1996; Sunquist and Sunquist 2002; Patterson 2004; Barnett et al. 2009) before their disappearance as part of the end-Pleistocene megafaunal mass extinction (Martin and Steadman 1999). Today, wild lions are found only in some parts of sub-Saharan Africa and at one locality in India, where they are confined mainly to protected areas such as national parks and game reserves. Even the relict populations found in these places seem to be declining at an alarming rate due to anthropogenic activities (Smuts 1978; Hanby and Bygott 1979; Nowell and Jackson 1996; Martin and Steadman 1999). Lions are today classified as vulnerable according to the IUCN Red List of Threatened Species (http://www.iucnredlist.org). The range collapse of lions in historical times has resulted in the extirpation of many marginal fragmented populations (O'Brien et al. 1987; Kingdon 1997).

All Pleistocene and modern day lions have been assigned to the genus *Panthera*, but with little consensus about the extent of overlap in their distribution (Barnett et al. 2009). Just like other big cats (e.g., leopard *Panthera pardus*, tiger *P. tigris*, jaguar *P. onca,* and snow leopard *P. uncia*), the lion displays several distinct phenotypic variations in body size, skull characteristics, coat color and thickness, retention of juvenile spots, and the presence or absence of mane in males. These marked characteristics may sometimes vary based on geographical regions (Hallgrimsson and Maiorana 2000; Mazak 2010). Many studies have employed the method of comparative analyses of craniometric data and morphometric analyses based on geographical regions to establish phylogenetic relationships between the lions (Sotnikova and Nikolskiy 2006; Mazak 2010). These analyses are then used in establishing distinctiveness between geographical regions (Hallgrimsson and Maiorana 2000; West and Packer 2002; Patterson 2004, 2007; Yamaguchi et al. 2004; Patterson et al. 2006). However, there can be complications with morphological identification sometimes due to the presence of shared primitive features (Sotnikova and Nikolskiy 2006), where morphological characteristics might not depict the true phylogeny of a species.

Different names have been proposed for the African lion based on geographical race (Meester and Setzer 1971). Currently, only two lion subspecies are recognized; the African lion (*P. leo leo*, Linneaus 1758) and the Asian lion (*P. leo persica*, Meyer 1826; O'Brien et al. 1987), and this has been supported by genetic studies (e.g., O'Brien et al. 1987; Driscoll et al. 2002; Burger et al. 2004; Dubach et al. 2005; Barnett et al. 2006). Many of these previous genetic studies did not have a good representation of lions from all over their range in West and Central Africa. In contrast, a recent phylogeographical study by Bertola et al. (2011), which was based on mitochondrial DNA sequences, had good representation of lions form West and Central Africa. Quite surprisingly, they found that Indian lions clustered with West and Central African lions forming a separate clade to lions in East and South Africa. Although their study had a good representation of lions from West and Central Africa, they did not include lions from some parts of West Africa – most notably Nigeria.

**Figure 1:**
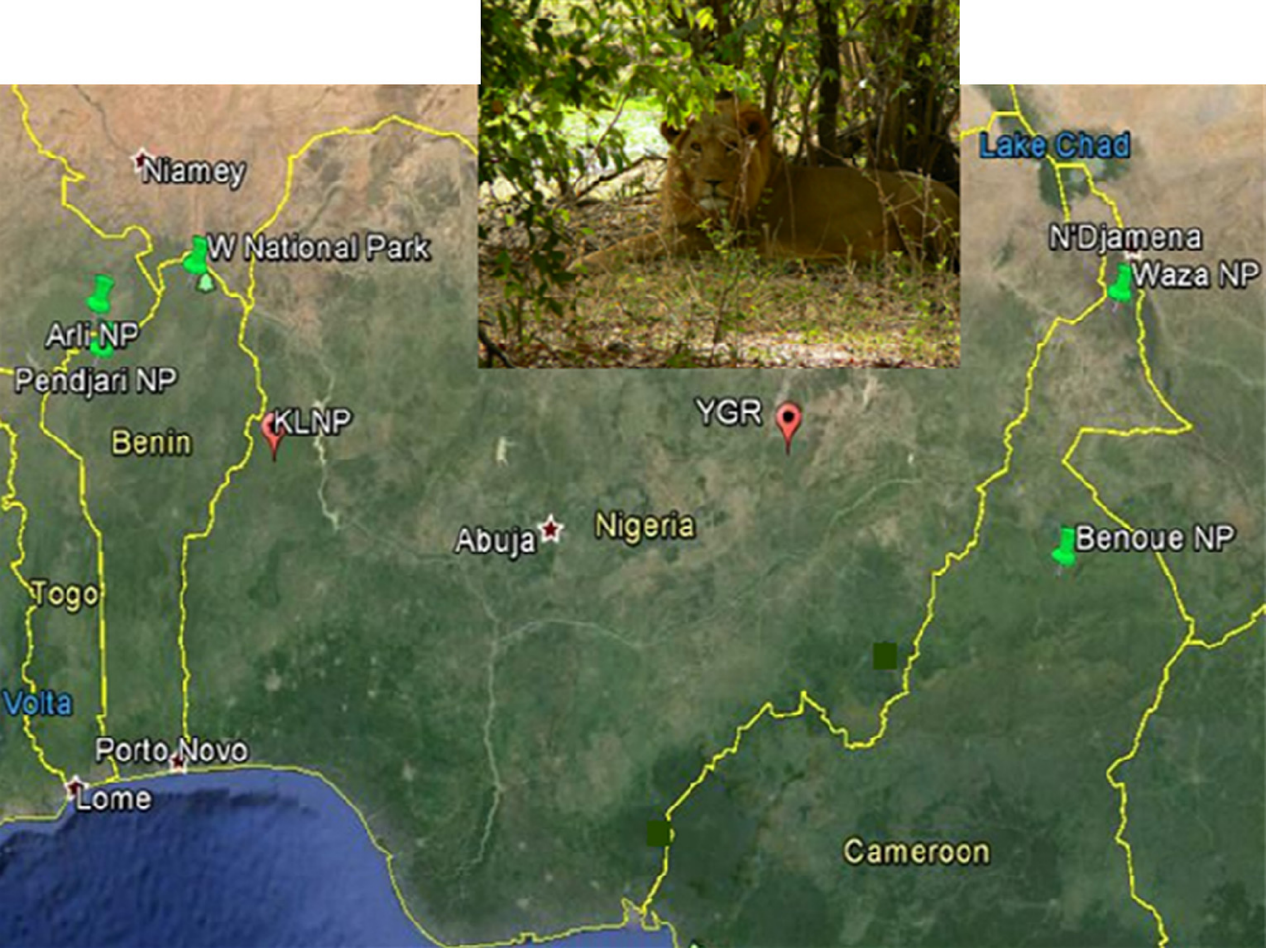
Locations of the two lion populations in Nigeria, KLNP and YGR (indicated by red markers), and neighboring lion populations in Benin and Cameroon (indicated by green markers).

In Nigeria, lions remain today in two isolated populations only; one in Kainji-Lake National Park in the western part of the country and one in Yankari Game Reserve in central northeast (Fig. 1). Inserted in the study map is a male lion that was sighted in Yankari Game Reserve.

The phylogenetic relationship between the lion populations in Nigeria and elsewhere in West and Central African is not yet fully understood. It is important to know, in the context of conservation, whether the lion populations in Nigeria form a monophyletic clade, or whether the two populations within Nigeria originate from different lines of ancestry. Describing the genetic makeup and phylogeographical history of endangered species is of general importance for understanding the evolutionary processes affecting the species and their geographical dynamics. This is, in turn, important for developing conservation strategies and making better informed management decisions (Mace et al. 2003).

A recent survey by Tende et al. (2014) used microsatellite analyses to determine the population size and level of gene flow within and between the lion populations in Nigeria. This study showed that the two remaining populations in Kainji-Lake National Park and Yankari Game Reserve exhibit signs of inbreeding and that they are genetically differentiated. In the present study, we aim to find out the relationship of the Nigerian lions to those from neighboring countries, as well as to lions in other parts of Africa, by phylogenetic analysis of the mitochondrial cytochrome *b* gene.

## Materials and Methods

Lion fecal DNA extracts from four different supposedly unrelated individuals from each of the study sites Yankari Game Reserve, central northeast (9°50′N and 10°30′E), and Kainji-Lake National Park, west Nigeria (09°55′N 03°57′E) were identified based on genotypes of nine microsatellite loci (Tende et al. 2014). Details of the DNA extraction protocol are given in Tende et al. (2010). Primers were designed to amplify three different segments covering most of the 1140 base pairs of the mitochondrial cytochrome *b* region. The primer sets for these regions are as follows: LCB1F (5′-TCACCGGCCTCTTTCTAGCCA-3′) and LCB1R (5′-AGGTGGACTGCTGCTAGGGCT-3′), LCB2F (5′-TCGGGGCCGACCTAGTAGAGTG-3′) and LCB2R (5′-TGGAAGTGTGGAGGGCAGGGA-3′), and LCB3F (5′-CCCGACAACTATACCCCCGCCA-3′) and LCB3R (5′-AGGGTACGCGTTCTCCTTTT-3′). All amplifications were carried out using a 2X Qiagen multiplex PCR kit in 10 μL reaction volume containing 5 μL Qiagen multiplex PCR buffer mix, 0.2 μmol/L forward primers (Applied Biosystems, Foster City, CA, USA), 0.2 μmol/L reverse primer, 2.6 μL of water, and 2 μL of DNA extract. A hot start at 95°C for 15 min with PCR profiles consisting of 35 cycles as follows: 90°C for 30 s; annealing temperature of 56°C for 30 s with elongation period of 72°C for 30 s. A blank control (reagents only) from extracted DNA process was included in all PCRs to monitor for contamination. The results of the PCR were evaluated by electrophoresis using 2% agarose gels and GelRed™ (Biotium, Hayward, CA) staining. Samples were further sequenced using the forward primers (BigDye sequencing kit; Applied Biosystems) in an ABI Prism® 3100 capillary sequencer (Applied Biosystems). Sequences were visually checked and aligned using Geneious vs.5.6.6 (Biomatters, Auckland, New Zealand) against 61 lion reference sequences from other parts of Africa and India downloaded from the GenBank.

The program MEGA5 (Tamura et al. 2011) was used to analyze the sequence data for the construction of phylogenetic trees. The substitution model for the construction of the tree was selected based on the lowest Bayesian information criterion (BIC). We identified the Hasegawa–Kishino–Yano (HKY) model as the best to describe the substitution pattern (Nei and Kumar 2000). The statistical confidence of each node was determined by assessing the frequency of nodes supported in 1000 bootstrap resampling of our data (Felsenstein 1985).

We used the maximum-likelihood approach with leopard (*P. pardus*) and tiger (*P. tigris*) as out-groups for the mitochondrial cytochrome *b* sequences to depict phylogenetic patterns among sequences. Using the program NETWORK v4.6.1.2 (http://www.fluxux-engineering.com/network_terms.htm), a haplotype network was generated for the sequences in order to gain additional insights into the relationships between Nigerian lion haplotypes and populations.

## Results

**Figure 2:**
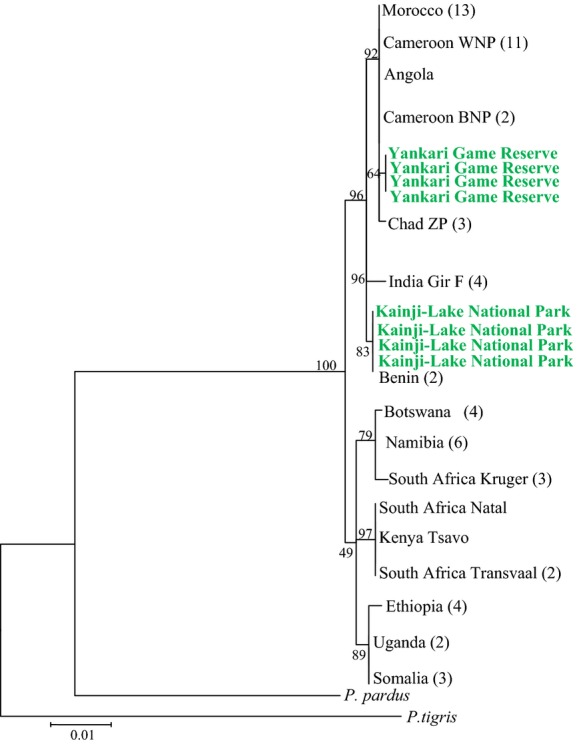
Phylogenetic tree from a maximum-likelihood analysis based on a set of lion mitochondrial cytochrome *b* sequences. Numbers in bracket represent the number of lion sequences downloaded from the GenBank for each area. Abbreviations are as follows: Cameroon Benoue National Park (Cameroon BNP), Cameroon Waza National Park (Cameroon WNP), Chad Zakouma National Park (Chad ZP), South Africa Transvaal (SA Transvaal), and South Africa Kruger National Park (SA Kruger). Highlighted in green are individuals from Yankari Game Reserve and Kainji-Lake National Park in Nigeria.

**Figure 3:**
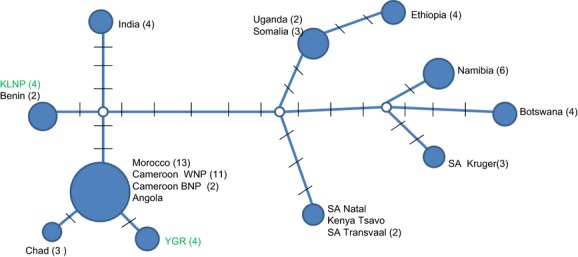
Haplotype network depicting genetic structure of lion within Nigeria and other parts of Africa and India. Abbreviations are as follows: South Africa Transvaal (SA Transvaal), South Africa Kruger National Park (SA Kruger), Yankari Game Reserve (YGR), and Kainji-Lake National Park (KLNP). Black lines indicate mutation events, and numbers in bracket indicate number of individuals used included in the analysis. Highlighted in green are individuals from Yankari Game Reserve and Kainji-Lake National Park in Nigeria.

We obtained sequences for all eight samples covering 944 bp of the cytochrome *b* gene. The analyzed samples showed no sequence variation within the two Nigerian study sites but differed by 0.4% between these sites. The cytochrome *b* phylogenetic analysis showed that the Nigerian lions cluster with 96% bootstrap support within the clade including lions from West and Central Africa and India (Fig. 2). Interestingly, the two Nigerian populations were phylogenetically separated: lions from Kainji-Lake National Park in western Nigeria grouped with lions in Benin (with 83% bootstrap support), whereas the population in Yankari Game Reserve in central northeastern Nigeria grouped with lions from Cameroon and a few other countries (92% bootstrap; Fig. 2). The haplotype network (Fig. 3) shows that the haplotype in the lion population in Yankari Game Reserve differs by two mutations from the common and widespread haplotype found in Morocco, Chad, Cameroon, and Angola. The haplotype found in Kainji-Lake National Park is identical to the haplotype of lions in Benin, differing by five mutations from the Yankari haplotype and by six mutations from the Indian haplotype (Fig. 3).

## Discussion

Using molecular data to reveal the distribution of genetic variation within and between populations can be a powerful means of enhancing our understanding of the evolutionary history of populations. For instance, Bertola et al. (2011) analyzed the cytochrome *b* gene and the control region of the lion from most parts of its range. They found that lions in West and Central Africa grouped with Asian lions rather than Southern and Eastern African populations. Our results, although restricted to the cytochrome *b* gene, are in agreement with their findings.

Our main finding is that lions from the two remaining populations in Nigeria do not cluster as sister taxa in phylogenetic mtDNA reconstructions. Instead, the cytochrome *b* gene of lions from Kainji-Lake National Park in western Nigeria is genetically similar to lions in Benin, whereas lions from Yankari Game Reserve in central northeastern Nigeria are more similar to the Cameroon lion population. This phylogeographical division probably reflects long-term separation due to a large geographical distance between these two localities and the lack of dispersal corridors for lions in Nigeria. In fact, the localities are geographically closer to populations in different neighboring countries; Benin in the case of KLNP and Cameroon in the case of YGR. The population in Kainji-Lake National Park may share a common history with lions in the nearby WAP complex, that is, the W and Pendjari National Parks in Benin, and Arly National Park in Burkina Faso (Fig. 1).

The clustering of the Nigerian lion with West and Central African lions as well as India shows their genetic as well as taxonomic distinctiveness as compared to East and Southern African lions. Thus, there might be a need to revise the currently recognized two lion subspecies (African lion *P. leo leo*, Linneaus 1758 and the Asian lion *P. leo persica*, Meyer 1826; O'Brien et al. 1987).

Recent surveys have shown drastic declines in population size and range of the lions (Packer et al. 2013). In Nigeria, it has been shown that the population is small with low genetic variability (Tende et al. 2014). As lions are increasingly confined to protected areas, the strategy for meta-population management will involve moving individuals between areas for population recovery and genetic reinforcement. The exchange of individuals between the two unique lion populations in Nigeria could help to enhance their separated gene pools. Also, translocation can be carried out between the remaining relict lion populations in Nigeria and neighboring countries of Benin and Cameroon when there is a need for that. Various studies have shown how natural exchanges of few individuals between populations have helped to enhance population growth and restore genetic diversity (Laikre and Ryman 1991; Vila et al. 2003; Liberg et al. 2004). For instance, low population size and genetic diversity were recorded among the Scandinavian wolves before the population was genetically rescued by a single immigrant from Finland (Vila et al. 2003). The arrival of this immigrant into the Scandinavian wolf population provided the possibility to avoid inbreeding, decrease the risk of inbreeding depression, and resulted in population growth. Also, laboratory and translocation experiments have indicated that small and inbred populations can be rescued by the contribution of minimal numbers of immigrants, helping to decrease inbreeding depression (Spielman and Frankham 1992; Westemeier et al. 1998; Madsen et al. 1999; Ebert et al. 2002; Vila et al. 2003), and bring about profound changes in genetic structures (Ball et al. 2000; Saccheri and Brakefield 2002; Vila et al. 2003). However, before initiating translocation programs, it is important to consider the genetic similarities between the target recipient population and alternative source populations in order to avoid introduction of genes from too distant populations.

### Dispersal difference in male and female lions

In vertebrates, mtDNA population genetic analyses are confined to tracing the migration patterns of maternal lineages, while analysis of the nuclear DNA inherited through both parents may give a complete picture of population structure inherited from both parents. Thus, being maternally inherited, mtDNA population genetic structures would reflect maternally directed natal-site fidelity and gene flow, whereas genome wide biparentally inherited nDNA assists in quantifying the levels of gene flow between subpopulations for both sexes. High rate of male-biased difference in dispersal patterns in lions (Pusey and Packer 1987) is expected to result in different distributions of genetic variation among populations for maternally (mtDNA) versus biparentally (nDNA) inherited molecular markers. The fixed differences in mtDNA between Kainji and Yankari lions suggest that female-mediated gene flow between the parks has been small or absent. Our study, based on microsatellite loci (nDNA) to investigate genetic differentiation between the two populations, also found substantial population structure (*F*_ST_ = 0.17) suggesting low levels of gene flow (Tende et al. 2014), with each population exhibiting significant signs of inbreeding (Yankari Game Reserve *F*_IS_ = 0.49) and (Kainji-Lake National Park *F*_IS_ = 0.38) (Tende et al. 2014). The suggested absence of female migration between our two study sites agrees with field studies demonstrating strongly restricted female dispersal in lions. Females can leave their natal pride to establish a new pride adjacent to their natal range, which often includes part of their old range, as compared to males that can disperse long distances from their natal range (Pusey and Packer 1987; Spong and Creel 2001). But whether the difference in mtDNA reflects overall differences in nuclear DNA in this study needs further investigation. The observed differences between the two populations could be due to phylogeography or genetic drift; a random change in allele frequency caused by chance event in small population sizes. The smaller the population size, the more likely genetic drift is to occur due to sampling errors.

## Conclusion and Conservation Perspectives

In summary, phylogenetic analysis of mitochondrial sequence data suggests that lions in Kainji-Lake National Park in western Nigeria are more closely related to lions in Benin, whereas lions in Yankari Game Reserve in central northeastern Nigeria are more closely related to lions in Cameroon. This difference reveals little or no female-mediated gene flow between the two Nigerian populations. As the finding by Tende et al. (2014) shows strong inbreeding levels in both populations, it is likely that mixing the two populations within Nigeria will have substantial benefits to both populations. Our finding is relevant for the management of the West and Central African lions and serves as an important guide for future conservation and management decisions. The study has provided an insight into the genetic composition of the lions in Nigeria in relation to the lion population in the neighboring countries of Benin and Cameroon. The exchange of individual lions between the two populations in Nigeria and with the neighboring countries will go a long way to boost their size as well as genetic status.
